# The role of dwelling type on food expenditure: a cross-sectional analysis of the 2015–2016 Australian Household Expenditure Survey

**DOI:** 10.1017/S1368980020002785

**Published:** 2021-06

**Authors:** Laura H Oostenbach, Karen E Lamb, Fiona Dangerfield, Maartje P Poelman, Stef Kremers, Lukar Thornton

**Affiliations:** 1 Department of Health Promotion, Faculty of Health, Medicine, and Life Sciences, Maastricht University, PO Box 616, Maastricht 6200, MD, The Netherlands; 2 Institute of Physical Activity and Nutrition, School of Exercise and Nutrition Sciences, Deakin University, Melbourne Burwood Campus, 221 Burwood Highway, Burwood, Melbourne 3125, Australia; 3 Melbourne School of Population and Global Health, University of Melbourne, Melbourne, Australia; 4 Consumption and Healthy Lifestyles Group, Wageningen University & Research, Wageningen, KN, The Netherlands

**Keywords:** Dwelling type, Apartment living, Food expenditure, Food purchasing, Australia

## Abstract

**Objective:**

To explore differences in proportion of food budget and total food expenditure by dwelling type.

**Design:**

A cross-sectional study using data from the Australian Bureau of Statistics 2015–2016 Household Expenditure Survey. Food expenditure was examined on multiple categories: fresh fruits, fresh vegetables, pre-prepared meals, meals in restaurants, hotels and clubs, and fast food and takeaway meals, using two-part models and zero-one inflated beta regression models. Dwelling types were categorised as separate house, semi-detached house, low-rise apartment and high-rise apartment.

**Setting:**

Australia, 2015–2016.

**Participants:**

Seven thousand three hundred and fifty-eight households from greater capital city areas.

**Results:**

Households living in high-rise apartments were estimated to allocate a greater proportion of their food budget to meals in restaurants, hotels and clubs, and to spend more (actual dollars) on that category, compared with other dwelling types. No substantial differences were estimated in the proportion of food budget allocated to the other food categories across dwelling types.

**Conclusions:**

The dwelling type households live in may play a role in their food budget. Households living in a high-rise apartment may potentially spend more on meals in restaurants, hotels and clubs than those living in other dwelling types. Given the growth in urban population and the changes in living arrangements, findings point to the critical need for a better understanding of the influence of dwelling types on food expenditure and call for research investigating the relationship between the two.

Food practices have changed in Australia and in other high-income countries over the past decades^(1,2)^. Home-prepared foods have increasingly been replaced by commercially prepared foods such as foods purchased from takeaway outlets^(2–5)^. Preparation and cooking time at home as well as expenditure on unprocessed foods including fresh fruit and vegetables, and fresh and frozen meat have declined^(2)^. Changes in food practices can be observed in food expenditure patterns^(6–8)^. To illustrate, Australian households spent 34 % of their total food expenditure on foods prepared away from home in 2016 compared with 26·4 % in 1999^(3)^. Trends towards away-from-home food consumption have also been observed elsewhere including the USA^(9)^. Behind these changes are substantial shifts in food production, processing and distribution systems as well as in food purchasing and eating opportunities^(10–12)^. Together, these changes have implications for the quantity and quality of foods consumed with data showing a trend towards higher intake of energy-dense, nutrient-poor foods and lower intake of fruits and vegetables^(13–15)^.

In Australia, changes in food expenditure coincide with a significant increase in obesity rates and an increase in rates of non-communicable diseases such as type 2 diabetes, CVD and several cancers^(13,16,17)^. The Australian Institute of Health and Welfare estimated that 1·4 % of the total burden of disease was independently attributable to low fruit intake, 1·2 % to low vegetable intake and 1·2 % to high processed meat intake^(18)^. This shows the urgency for public health professionals to identify better ways to improve population nutritional status^(19,20)^.

Further, the changes observed have been paralleled by a growth in urban living. The percentage of the world’s urban population has surged, with 54 % living in cities in 2015. This number is projected to further increase to 60 % in 2030 and to reach 66 % by 2050^(21)^. Cities have grown to accommodate more people resulting in higher density living^(22)^. Consequently, living arrangements have experienced a transformation and apartment living is becoming ever more prevalent^(23,24)^. In Australia, the number of apartments has increased by 78 % over the past two decades and there is currently about one apartment for every five residential houses^(23)^. Families with children represent 44 % of all families living in apartments in Australia^(23)^.

The shift to higher density living represents changes in material infrastructures, including dwelling design. Apartments are generally smaller than residential detached houses^(25)^. This shift may therefore represent lifestyle changes for many dwellers^(26)^. Previous research suggests high-rise apartment living is associated with a weaker sense of community^(27–29)^, reduced social interaction^(29–32)^ and more behaviour problems among children^(27)^. There is limited evidence on the impact of dwelling type on food expenditure and food practices more generally. A Canadian study examined food behaviours in relation to apartment living; however, the sample in the current study was restricted to single men living in Montreal^(33)^. While convenience and budget were found to be the main drivers of food choices^(33)^, it is difficult to draw broader conclusions from that study given the characteristics of the sample, the small sample size and location.

Within the social practice theory, houses and apartments are not merely buildings but they are part of the domestic infrastructure of the home, playing a role in social practices such as food practices (including planning, purchasing, preparation and consumption of food)^(26,34)^. The social practice theory suggests food practices are performed through the interplay of materials (e.g., space to cook, space to eat, proximity to restaurants and other food outlets), meanings (i.e., shared understandings and expectations about why, how, when and with whom to eat) and competences (e.g., skills on how to prepare and eat food). Changes in one of the three elements can modify the other elements, engendering changes within food practices themselves. Different dwelling types may alter available materials, leading to changes in meanings and competences attached to food practices^(26,34)^.

Research on built environments and health is increasingly finding their way into urban design and land use planning policies, yet the impact of the dwelling type is largely unknown. Although the growth in apartment living is concomitant with changes in food expenditure^(3,23)^, the direct link between apartment living and household food expenditure remains under-investigated and is the primary aim of the current study. It is argued that apartment living may influence food expenditure through internal design factors such as restricted kitchen size, easier access to convenience foods or through social norms. In the absence of other quantitative data related to links between apartment living and food expenditure, the current study provides an important first step for putting this topic on both the research and policy agenda. Specifically, the current study could inform further research investigating the role of the home infrastructure and high-density urban living on food behaviours.

## Methods

### Data source

The study used data from the 2015–2016 Australian Household Expenditure Survey (HES) conducted by the Australian Bureau of Statistics (ABS). The HES is a nationally representative survey gathering data on weekly household expenditure on a wide range of items relating to goods and services such as food, transport and health care and is conducted by the ABS every 6 years^(36)^. ABS sampled households living in private dwellings from urban and rural areas of Australia. Data were collected only from usual residents, that is, residents who considered the dwelling as their main or own home. Households were selected through a stratified, multistage cluster design from the Australian Private Dwelling Framework of the Population Survey Master Sample, covering 97 % of the Australian population^(37,38)^. Further, to ensure that results were representative of income and expenditure patterns across a whole year, ABS distributed selections across a 12-month period^(38)^. Households were excluded if they failed to respond, responded inadequately or if no contact could be made. The final HES sample included 10 046 households^(38)^.

### Data collection instruments

The current study used HES data on dwelling types and weekly household expenditure on food items. Dwelling type and household characteristics data were collected by trained ABS interviewers using a computer-assisted questionnaire^(39)^. Food expenditure data were collected using a 2-week expenditure diary completed by every household member aged 15 years or over. Respondents had to cite the type of store, describe the food item and report the amount they paid^(40,41)^. Foods purchased were categorised into one of 131 food expenditure categories. Weekly household food expenditure for each food category was then calculated by summing all reported expenditure for all household members and dividing this by the number of weeks in the reporting period, that is, 2 weeks^(42)^. This method resulted in weekly household expenditure variables.

### Sample

**Fig. 1:**
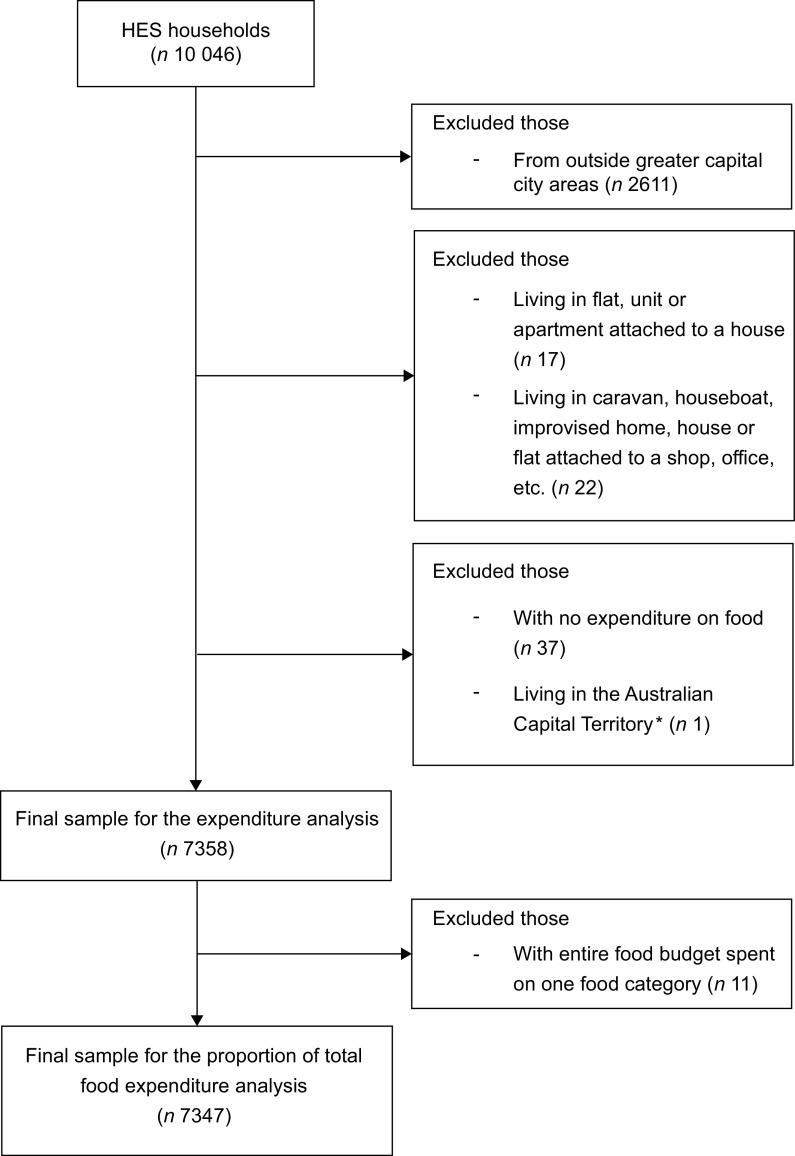
Flowchart of households included in the analysis. ^*^ Household Expenditure Survey (HES) sample included 294 households from the Australian Capital Territory but only one was classified as living in greater capital city areas. All respondents from the Northern Territory were excluded as they were all classified as living outside greater capital city areas

Given the purpose of the current study, the sample only included households either living in a: separate house; semi-detached, row or terrace house, townhouse, etc. with one storey; semi-detached, row or terrace house, townhouse, etc. with two or more storeys; flat, unit or apartment in a one or two storey block; flat, unit or apartment in a three storey block; or flat, unit or apartment in a four or more storey block. These dwelling type categories were combined into four categories for analysis: (1) separate house; (2) semi-detached, row or terrace house, townhouse, etc. with one or more storeys, termed semi-detached house; (3) flat, unit or apartment in a one, two or three storey block, termed low-rise apartment and (4) flat, unit or apartment in a four or more storey block, termed high-rise apartment. As the growth in apartment living is primarily an urban phenomenon^(43)^, the sample only included households living in greater capital city areas^(44)^. The final sample of the current study included 7358 households for the weekly household food expenditure analysis and 7347 for the proportion of total weekly household food expenditure analysis. Figure 1 provides a flowchart of households included in the study.

### Outcome variables: food item categories

The study examined weekly household food expenditure, measured in Australian dollars (AUS$), and proportion of total weekly household food expenditure spent on five categories of food expenditure items. These categories included: (1) fresh fruits; (2) fresh vegetables; (3) pre-prepared meals; (4) meals in restaurants, hotels and clubs and (5) fast food and takeaway. The categories chosen can be seen as proxies for healthier and unhealthier food options and may also reflect food behaviour choices (e.g., eating out *v*. preparing foods at home). Table 1 describes food items included in each expenditure category. The study included ten outcome variables, that is, a proportion and an expenditure indicator for each of the five food categories. In addition, the study examined the number of households with or without any expenditure on the different categories of food items.

**Table 1:** Items included in each food expenditure category

Food categories	Foods included in the category
Fresh fruits	Fresh fruits including citrus fruit, stone fruit, apples, pears, berries, grapes, melons, tropical fruit and bananas
Fresh vegetables	Fresh vegetables including potatoes, onions, other root vegetables, tomatoes, capsicum, cucumber, zucchini, flower vegetables, leaf vegetables, peas, beans, pumpkin and mushrooms
Pre-prepared meals	Frozen prepared meals and packaged prepared meals
Meals in restaurants, hotels and clubs	Examples include bistro meal, breakfast out, Chinese meal out, counter lunch, lunch out, dinner out, meal (restaurant), meals out, pizza (eat in), smorgasbord
Fast food and takeaway	Examples include McDonalds (eat in or takeaway), pizza (takeaway), Kentucky Fried Chicken, Dagwood dog, Indian food (takeaway), kebab (takeaway), giros (takeaway), sausage roll (not frozen), pork pies (not frozen), nachos (takeaway), baklava (cake)

### Confounders

Potential confounders were identified and defined at household level. These included household composition (lone male, lone female, one person aged 15 or older with at least one child younger than 15 years (n.b. children younger than 15 years are defined by the ABS as dependent), two people aged 15 or older without children younger than 15 years, two people aged 15 or older with at least one child younger than 15 years, three or more people aged 15 or older without children younger than 15 years, three or more people aged 15 or older with at least one child younger than 15 years), tenure type (owner with a mortgage, owner without a mortgage, renter and other), state/territory of residence (New South Wales, Victoria, Queensland, Western Australia, South Australia and Tasmania), age of the household reference person (years) and weekly household income (AUS$).

### Statistical analyses

Descriptive statistics were examined for all outcome and potential confounder variables across dwelling types (see online supplementary material, Additional file 1). The total expenditure and proportion of weekly food expenditure for each food category are presented for all households, as well as only for households that had some expenditure for the given food category. Medians and 25th and 75th percentiles are presented for proportions and expenditure due to the highly skewed nature of the outcome distributions.

As proportions lie in the closed unit interval [0;1] and many households had a proportion of total weekly food expenditure equal to zero, zero-inflated beta regression models were used to assess the association between dwelling types and proportion of total weekly household expenditure on each food category^(46)^. Unlike beta regression models, which only model values between 0 and 1, zero-one inflated beta (zoib) regression accounts for mass points at 0 and 1, assuming that proportions of 0 or 1 occur through a different process from that of the other proportions^(47–49)^. Typically, zoib contains three parts: two separate logistic regression models to predict whether the proportion equals 0 (zero-inflate) or 1 (one-inflate), and one beta regression model to predict proportions in the open unit interval (0;1)^(49)^. As only eleven households had a proportion equal to 1 for a given food category (i.e., households spent their entire food budget on one food category) (see online supplementary material, Additional file 2), these were excluded from the analysis of proportions as there were insufficient numbers to model the one-inflate part. Model coefficients were exponentiated to obtain predicted odds ratios (OR) of observing a zero proportion (i.e., zero expenditure on that food category) and relative proportion ratios (i.e., the factor by which the relative proportion ratio changes^(50)^). The mean proportions spent on each food category across dwelling types were estimated from the combined parts.

A large proportion of households had no expenditure for given food categories. Therefore, two-part regression models^(45)^ were used to assess the association between dwelling types and total weekly household expenditure on each food category. The first part of the regression models examines the probability of having any expenditure for the food category considered, while the second part models the amount spent on the category, given that the household had reported spending money on that food category. Logistic regression was used for the first (binary) part and log-linear regression was used for the second (continuous) part to account for the skewed nature of the distribution of expenditure. Results from the analysis were exponentiated to obtain estimates of the OR for the first part and geometric means for the second part. Both models were used to estimate marginal mean expenditures for each food category across dwelling types. Standard errors for the marginal effects were estimated via bootstrapping by drawing 1000 random samples with replacement and selection at household level.

All analyses were conducted in 2019 using the statistical software package STATA version 15.1. The user-contributed commands *zoib*^(46)^ and *twopm*^(45)^ were used to fit the zoib and two-part regressions. Models were adjusted for potential confounders including household composition, tenure type, state/territory, age of the reference person and weekly household income.

## Results

Table 2 shows the percentage of households without any expenditure as well as the total and proportion of total weekly household expenditure for each food category across dwelling types. The highest percentage of households not spending any money on fresh fruits and vegetables was households living in a low-rise apartment (*n* 131, 22·6 % for fresh fruits; *n* 103, 17·8 % for fresh vegetables). Expenditure on meals in restaurants, hotels and clubs also represented the highest median proportion of total weekly food expenditure (32·9 %) among those living in a high-rise apartment with any expenditure on the category. Similar patterns were observed when considering the sample as a whole. Among only those with some expenditure on these food categories, households living in a separate house had the highest weekly median expenditure on fresh fruits ($9·3) and vegetables ($11·1). However, among those with some expenditure on meals in restaurants, hotels and clubs, households living in a high-rise apartment reported the highest weekly median expenditure with $65·1 spent on the category. Sample characteristics by dwelling type and potential confounders are presented in online supplementary material, Additional file 1.

**Table 2:** Descriptive statistics (unadjusted) on the proportion of total weekly food expenditure and weekly household expenditure for each food expenditure category across dwelling types

	Separate house				Semi-detached house				Low-rise apartment				High-rise apartment			
	*n*	%	Median	p25, p75	*n*	%	Median	p25, p75	*n*	%	Median	p25, p75	*n*	%	Median	p25, p75
Sample size*	5609	76·2			934	12·7			579	7·9			236	3·2		
No expenditure*^,^†																
Fresh fruits	628	11·2			155	16·6			131	22·6			37	15·7		
Fresh vegetables	520	9·3			137	14·7			103	17·8			30	12·7		
Pre-prepared meals	2290	40·8			452	48·4			304	52·5			134	56·8		
Meals in restaurants, hotels and clubs	1994	35·6			349	37·4			235	40·6			56	23·7		
Fast food and takeaway	1194	21·3			212	22·7			157	27·1			53	22·5		
Sample size for proportion analysis (*n* 7347)	5605	76·3			932	12·7			575	7·8			235	3·2		
Proportion of total weekly food expenditure among all participants‡																
Fresh fruits			0·042	0·018, 0·077			0·039	0·012, 0·077			0·037	0·008, 0·073			0·035	0·012, 0·067
Fresh vegetables			0·052	0·026, 0·085			0·049	0·019, 0·087			0·044	0·015, 0·084			0·044	0·018, 0·086
Pre-prepared meals			0·010	0·000, 0·032			0·005	0·000, 0·032			0·000	0·000, 0·030			0·000	0·000, 0·023
Meals in restaurants, hotels and clubs			0·083	0·000, 0·223			0·095	0·000, 0·272			0·088	0·000, 0·274			0·240	0·030, 0·465
Fast food and takeaway			0·079	0·018, 0·162			0·074	0·016, 0·163			0·089	0·000, 0·195			0·087	0·018, 0·174
Proportion of total weekly food expenditure among those with expenditure only‡																
Fresh fruits			0·048	0·025, 0·081			0·049	0·026, 0·084			0·051	0·027, 0·091			0·044	0·020, 0·078
Fresh vegetables			0·057	0·033, 0·089			0·060	0·031, 0·094			0·057	0·030, 0·093			0·049	0·026, 0·090
Pre-prepared meals			0·027	0·013, 0·052			0·031	0·016, 0·058			0·033	0·016, 0·062			0·026	0·014, 0·051
Meals in restaurants, hotels and clubs			0·176	0·092, 0·300			0·222	0·113, 0·359			0·238	0·130, 0·394			0·329	0·195, 0·495
Fast food and takeaway			0·109	0·054, 0·190			0·109	0·055, 0·202			0·140	0·074, 0·235			0·115	0·060, 0·201
Sample size for expenditure analysis (*n* 7358)	5609	76·2			934	12·7			579	7·9			236	3·2		
Total weekly expenditure among all participants ($AUS)*																
Fresh fruits			7·9	2·8, 15·7			5·7	1·8, 12·7			4·6	0·8, 10·7			6·3	1·5, 12·1
Fresh vegetables			9·9	4·2, 17·7			7·2	2·5, 14·7			5·8	1·5, 12·1			6·9	2·8, 15·2
Pre-prepared meals			2·0	0·0, 6·8			1·0	0·0, 5·4			0·0	0·0, 4·0			0·0	0·0, 3·8
Meals in restaurants, hotels and clubs			15·2	0·0, 52·2			12·5	0·0, 51·0			9·8	0·0, 43·8			38·2	3·2, 104·7
Fast food and takeaway			14·6	2·9, 38·1			11·3	2·1, 29·7			10·7	0·0, 31·9			14·7	2·7, 33·9
Total weekly expenditure among those with expenditure only ($AUS)*																
Fresh fruits			9·3	4·4, 17·1			7·5	3·5, 14·1			6·9	3·5, 12·4			8·0	3·3, 13·5
Fresh vegetables			11·1	5·8, 18·8			9·1	4·3, 16·6			8·0	3·8, 13·2			8·1	4·5, 16·4
Pre-prepared meals			5·5	2·9, 10·4			5·0	2·5, 9·5			4·4	2·5, 8·7			4·4	2·5, 9·1
Meals in restaurants, hotels and clubs			37·2	16·9, 78·8			35·2	15·3, 89·2			35·9	16·3, 73·3			65·1	27·8, 125·6
Fast food and takeaway			22·7	9·0, 46·5			17·5	7·2, 39·1			19·5	8·8, 42·7			23·4	8·9, 41·2

* Analyses run on expenditure sample.

† Column percentage.

‡ Analyses run on proportion sample.

### Dwelling type and proportion of total weekly food expenditure

**Fig. 2:**
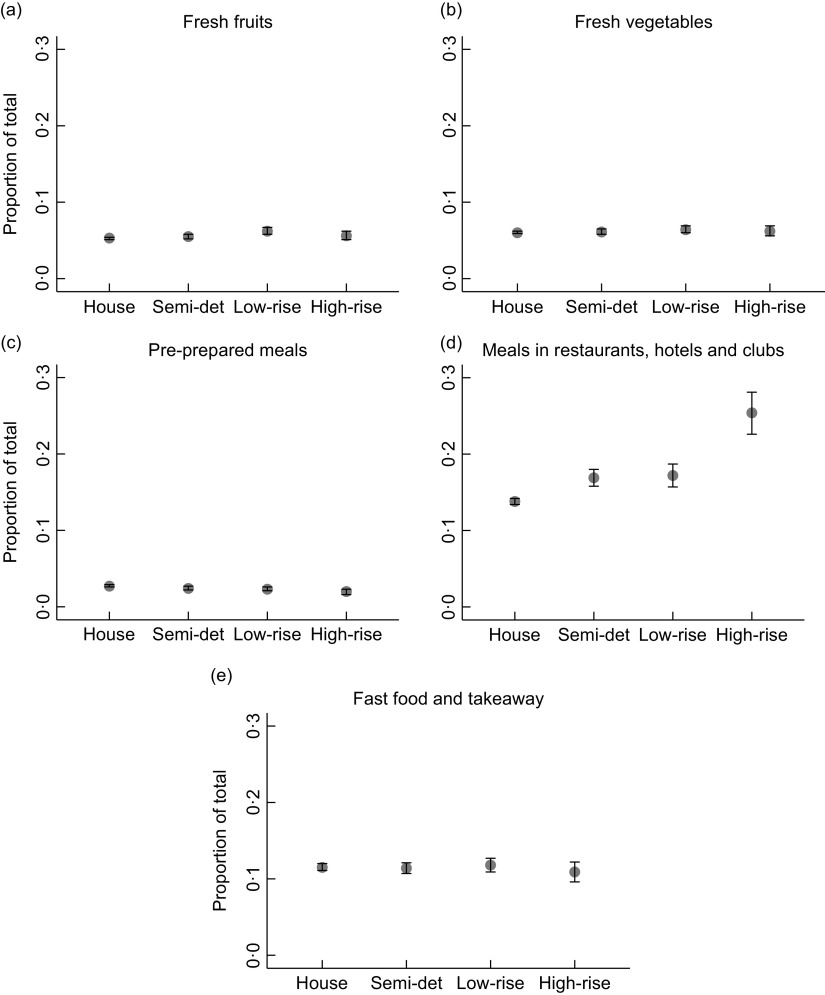
Estimated proportion of total weekly household food expenditure on each food category across dwelling types adjusted for potential confounders including household composition, tenure type, state/territory, age of the reference person and weekly household income. (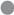), Estimated mean proportion of total; (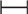), 95 % confidence interval

As the zoib regression models provided separate results for the zero-inflate and beta regression parts, only marginal effects of combined parts are reported (Fig. 2). The estimated average proportion of total food expenditure spent on fresh fruits and fresh vegetables was similar across dwelling types (Fig. 2a and 2b). Out of the five food categories, pre-prepared meals was the food category on which all dwelling types were estimated to spend the smallest proportion of their total food expenditure, with less than 3 % across dwelling types (Fig. 2c). All dwelling types were estimated to spend more than 13 % of their total food expenditure on meals in restaurants, hotels and clubs. Those living in a separate house were estimated to, on average, spend the smallest proportion of total food expenditure on meals in restaurants, hotels and clubs (0·138, 95 % CI = 0·134–0·142), whereas those in a high-rise apartment spent the highest proportion (0·254, 95 % CI = 0·226–0·281) (Fig. 2d). When considering fast food and takeaway meals, all dwelling types were estimated to spend more than 10 % of their total food expenditure on that category. However, the difference in the estimated average proportion of total food expenditure spent on fast food and takeaway meals across dwelling types was less noticeable than that spent on meals in restaurants, hotels and clubs (Fig. 2e). The OR of observing zero spending and the relative proportion ratios (for those who had some expenditure) for each food category are presented in online supplementary material, Additional file 3. The estimated average proportion of total food expenditure spent on each food category across dwelling types is presented in online supplementary material, Additional file 4.

### Dwelling type and weekly food expenditure

**Fig. 3:**
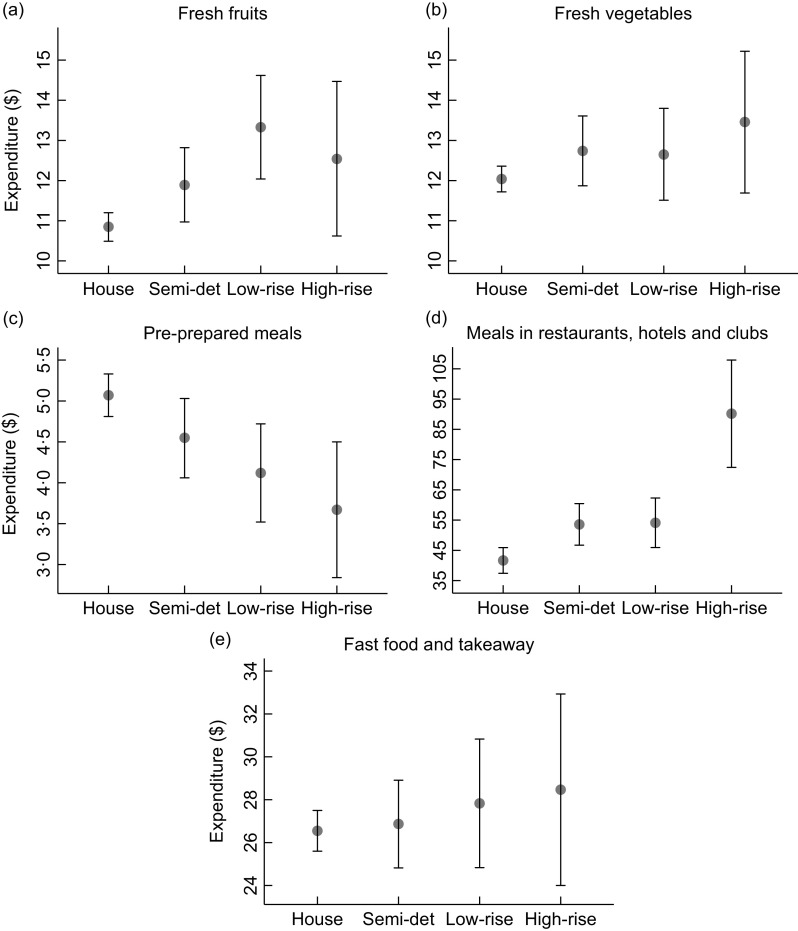
Estimated weekly household expenditure on each food category across dwelling types adjusted for potential confounders including household composition, tenure type, state/territory, age of the reference person and weekly household income. (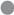), Estimated weekly mean expenditure; (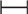), 95 % confidence interval. Note that different scales are used between graphs

As the two-part regression models provided results for the first and second part separately, only marginal effects of combined parts are reported (Fig. 3). The average fresh fruit expenditure was estimated to be greater for households living in a low-rise apartment ($13·33, 95 % CI = 12·04–14·62) compared with households living in a separate house ($10·85, 95 % CI = 10·49–11·20). The difference in the estimated average fresh vegetables expenditure was less noticeable across dwelling types than that in the estimated average fresh fruit expenditure (Fig. 3a and 3b). When it comes to pre-prepared meals, households living in a separate house were estimated to spend, on average, more on that food category compared with households living in a high-rise apartment ($5·07, 95 % CI = 4·81–5·33 *v*. $3·67, 95 % CI = 2·84–4·50) (Fig. 3c). The average expenditure on meals in restaurants, hotels and clubs was estimated to be greater for households living in a high-rise apartment ($90·17, 95 % CI = 72·43–107·90) compared with all other dwelling types, with the lowest expenditure observed among those in a separate house ($41·66, 95 % CI = 37·42–45·90) (Fig. 3d). The estimated average expenditure on fast food and takeaway meals varied less across dwelling types as compared with that on meals in restaurants, hotels and clubs (Fig. 3d and 3e). The odds of spending any money and the geometric mean expenditure (for those who had some expenditure) on each food category are presented in online supplementary material, Additional file 5. The estimated average expenditure on each food category across dwelling types is presented in online supplementary material, Additional file 4.

## Discussion

The current study aimed to investigate whether there were differences in the proportion of food budget households allocate to different food categories given the type of dwelling they live in. It also examined total food expenditure. Results indicated that households living in a high-rise apartment spend more on meals in restaurants, hotels and clubs than those living in other dwelling types and that this expenditure consumes a greater proportion of their entire food budget. Specifically, households living in a high-rise apartment spend over 10 % more of their food budget on meals in restaurants, hotels and clubs compared with households living in a separate house. Potential factors may relate to apartment designs which are often smaller^(25)^, with potentially less space to store food and prepare, cook and consume meals. Limited space might discourage residents from cooking and eating at home, or having friends or family over for meals^(32)^. Households may seek foods prepared and eaten away from home to compensate for the limited space available in their homes. Another related explanation may be the social aspect of eating out in restaurants, hotels and clubs. Previous studies have found that apartment living may be associated with a lower sense of community^(27–29)^ and lower social interaction^(29–32)^. Households living in apartments may be more likely to go out for meals in restaurants, hotels and clubs to engage in social interaction as the design of their homes and kitchen may not foster social bonds and intimacy among friends and family^(32)^. From a health perspective, this is of concern as out-of-home foods are often unhealthy and tend to be of lower nutritional quality and are associated with a higher total energy intake compared with home-prepared foods^(51)^. An increased expenditure on meals in restaurants, hotels and clubs could therefore have detrimental consequences for overall diet and health status, highlighting the need to reduce barriers to home cooking.

No discernible difference was observed across dwelling types for expenditure and proportion of total food expenditure on fast food and takeaway meals. One plausible explanation is that takeaway meals are easily accessible to every household regardless of where they live or that time is a more important determinant of this fast food use than factors related to dwelling type.

Furthermore, the results of the present study showed that while all households may, on average, spend a rather similar proportion of their food budget on fresh fruits and fresh vegetables, households living in a low-rise apartment spend $2·48 more than those living in a separate house on fresh fruits per week. This may be considered a small difference; however, $2·48 represents approximately three bananas, four apples, four kiwis or even ten mandarins per week^(53)^. This difference may be explained by the likelihood that households living in apartments are located in areas with easier access to supermarkets, greengrocers or markets, and therefore, this more frequent exposure to fresh fruits and fresh vegetables could increase impulse-driven (unplanned) purchases^(54–56)^. In contrast, households living in a separate house may have more space to store food and therefore engage in grocery shopping on a less frequent basis resulting in less purchasing of fresh fruits and fresh vegetables due to the perishable nature of these foods. While only fresh fruit and vegetable items were considered in the analysis presented, further analysis included a more comprehensive measure of fruits and vegetables that captured additional items such as frozen or canned fruits and vegetables, or dried fruits, and again no major difference was found (results not shown).

Interestingly, results showed that households living in a separate house spent more on pre-prepared meals compared with households living in a high-rise apartment. This might reflect time scarcity experienced by those living in a separate house (perhaps as a result of living further away from major cities and having increased commute time) as well as reduced food store availability. Households living in a separate house are likely to be located in areas with lower-population density and less levels of commercialisation; thus, they may have fewer food opportunities compared with those living in apartments. Previous studies indicated that limited time^(57–59)^ and the perishable nature of fresh foods may be barriers to home cooking^(59,60)^. For households living in a separate house, pre-prepared meals can provide a solution to these barriers by offering shelf-stable meals that can easily and quickly be eaten at home.

### Strengths and limitations

The study is strengthened by utilising data from a nationally representative large sample and by the comprehensive data collection methodology. Appropriate regression modelling approaches were employed to deal with the nature of expenditure data (i.e., high number of zero values and skewed distribution) and the analysis was able to control for a number of key confounders. Further, although the reporting period was 2 weeks, ABS sampled across a 12-month period, ensuring that results were representative of income and expenditure patterns across a whole year^(38)^. Nevertheless, results must be interpreted in the context of the following limitations. Food expenditure is a combination of quantity and price. These findings may not directly reflect the quantity or quality of foods being consumed by households. For example, higher expenditure on a food category may reflect either greater quantities or more expensive options. Additionally, the study was not able to account for food waste, meaning that although food was purchased (e.g., fresh fruit), whether it was consumed is unknown. Nonetheless, household expenditure data may be useful to explore trends and patterns of behaviours across dwelling types. Other studies have also used expenditure data to examine tobacco use^(61–63)^, gambling^(62,64,65)^, alcohol purchasing^(62,66)^ and food purchasing^(2,62,67)^. This type of data can be an important tool to inform and develop health strategies and policies in various areas^(68–70)^. Further limitations include the fact that only five categories of food expenditure were considered. Those living in high-rise apartments were estimated to spend considerably more on meals in restaurants, hotels and clubs compared with those living in other dwelling types. The proportion of total food expenditure was also estimated to be higher for that category. This suggests that another food category would have had to be substituted for. However, the other food categories considered gave no indication of what those households spent less on. Further research is required on overall food expenditure patterns of those in high-rise apartments. High-rise apartments were defined as apartments with four or more storeys; therefore, the current study was unable to differentiate between buildings of four storeys and larger buildings. With the increase in apartment living in Australia, the ABS and other studies should consider including additional categories related to apartment housing types. Reporting bias and recall bias can also not be ruled out as expenditure data relied on the accuracy of self-report. Some respondents may have over-reported buying food items they believed to be more socially desirable, while some others may have under-reported food items they believed to be less socially desirable. Respondents may also have inaccurately and incompletely reported all the food items they bought. This may pose a threat to the internal validity of the study^(71)^. However, as the HES gathered data not only on food expenditure but also on a wide range of other items^(36)^, respondents may not have had the time to consider what spending would be socially desirable. ABS data collection methods also minimised these risks by asking for receipts where possible^(41)^. Another important limitation to acknowledge is that data on food and living preferences are missing. Perhaps preferences for living near various food options, eating out often or socialising may lead individuals to deliberately choose to live in areas with greater commercialisation and high-rise apartments. Thus, apartments may by default become the dwelling type they live in.

### Implications and recommendations for further research

Although changes in food expenditure are paralleled by the growth in apartment living^(2,3,23)^, no previous study has explored the relationship between dwelling types and food expenditure. The current study is the first to shed light on this potential relationship, with results showing that dwelling types may play a role in food expenditure. These findings are of great significance as cities are adopting compact city planning strategies to manage urban densification and population growth, increasing the prevalence of apartment living^(24,72)^. These results may serve as a starting point for further research examining how the home infrastructure, particularly that of apartments (e.g., limited food storage, cooking space, cooking facilities and eating areas), may influence food expenditure. Further research is required in other (international) contexts to build a body of evidence upon which policy recommendations can be made. Opportunities also exist to investigate whether differences are also reflected in measures of food consumption. Understanding the influence of dwelling type and design on food expenditure and consumption may allow for the development of context-dependent public health interventions aiming at improving population nutritional status. Additionally, further research may help provide guidance on how apartments and environments surrounding apartments should be designed to enable healthy food practices.

## Conclusions

The current study explored differences in how much households spend on different food categories depending on the type of dwelling they live in. The fact both the total amount of dollars spent and the proportion of the total household food expenditure on meals out were greater among those in high-rise apartments leads us to believe that food behaviours differ among this group and that dwelling type is likely to play a part. Whether this is through the characteristics of those living in apartments, their surrounding environment or barriers faced by the internal design of their apartments should be explored in future research.

## Supplementary material

For supplementary material accompanying this paper visit https://doi.org/10.1017/S1368980020002785. click here to view supplementary material
