# Supplementary material for: The role of dwelling type on food expenditure: a cross-sectional analysis of the 2015–2016 Australian Household Expenditure Survey
Source: Public Health Nutr. 2020 Aug 24;24(8):2132–43. doi: 10.1017/S1368980020002785 (PMC8145465; doi:10.1017/S1368980020002785)
Supplement: Supplementary file 1 [file S1368980020002785sup.zip › S1368980020002785sup001.docx]

**Additional file 1:** Sample characteristics by dwelling type and model confounders

|  | Separate house | | | Semi-detached house | | | Low-rise apartment | | | High-rise apartment | | |
| --- | --- | --- | --- | --- | --- | --- | --- | --- | --- | --- | --- | --- |
|  | n | % | | n | % | | n | % | | n | % | |
| Total (N=7358) ^a^ | 5609 | 76.2 | | 934 | 12.7 | | 579 | 7.9 | | 236 | 3.2 | |
| Household Composition ^b^ |  |  | |  |  | |  |  | |  |  | |
| Lone, male | 464 | 8.3 | | 156 | 16.7 | | 151 | 26.1 | | 55 | 23.3 | |
| Lone, female | 762 | 13.6 | | 255 | 27.3 | | 164 | 28.3 | | 49 | 20.8 | |
| One person aged >15 years, at least one child <15 years | 204 | 3.6 | | 44 | 4.7 | | 27 | 4.7 | | 5 | 2.1 | |
| Two people aged >15 years, no children <15 years | 2140 | 38.1 | | 291 | 31.2 | | 167 | 28.8 | | 81 | 34.3 | |
| Two people aged >15 years, at least one child <15 years | 928 | 16.5 | | 106 | 11.3 | | 45 | 7.8 | | 32 | 13.6 | |
| Three or more people aged >15 years, no children <15 years | 804 | 14.3 | | 60 | 6.4 | | 19 | 3.3 | | 10 | 4.2 | |
| Three or more people aged >15 years, at least one child <15 years | 307 | 5.5 | | 22 | 2.4 | | 6 | 1.0 | | 4 | 1.7 | |
| Tenure type ^b^ |  |  | |  |  | |  |  | |  |  | |
| Owner without a mortgage | 2334 | 41.6 | | 252 | 27.0 | | 73 | 12.6 | | 47 | 19.9 | |
| Owner with a mortgage | 2041 | 36.4 | | 191 | 20.4 | | 75 | 13.0 | | 42 | 17.8 | |
| Renter | 1129 | 20.1 | | 473 | 50.6 | | 425 | 73.4 | | 140 | 59.3 | |
| Other | 105 | 1.9 | | 18 | 1.9 | | 6 | 1.0 | | 7 | 3.0 | |
| State or Territory ^b, c^ |  |  | |  |  | |  |  | |  |  | |
| New South Wales | 1152 | 20.5 | | 257 | 27.5 | | 237 | 40.9 | | 115 | 48.7 | |
| Victoria | 1503 | 26.8 | | 292 | 31.3 | | 124 | 21.4 | | 67 | 28.4 | |
| Queensland | 817 | 14.6 | | 109 | 11.7 | | 84 | 14.5 | | 19 | 8.1 | |
| South Australia | 840 | 15.0 | | 120 | 12.8 | | 58 | 10.0 | | 14 | 5.9 | |
| Western Australia | 810 | 14.4 | | 128 | 13.7 | | 43 | 7.4 | | 17 | 7.2 | |
| Tasmania | 487 | 8.7 | | 28 | 3.0 | | 33 | 5.7 | | 4 | 1.7 | |
|  |  |  | |  |  | |  |  | |  |  | |
|  | Median | P25 | P75 | Median | P25 | P75 | Median | P25 | P75 | Median | P25 | P75 |
| Age of the reference person (years) | 56.0 | 43.0 | 69.0 | 52.5 | 37.0 | 69.0 | 45.0 | 32.0 | 64.0 | 40.0 | 31.0 | 62.0 |
| Weekly household income ($AUS) | 1771.9 | 1209.0 | 2544.2 | 1306.1 | 911.3 | 1992.3 | 1104.9 | 813.6 | 1732.6 | 1443.2 | 921.3 | 2208.2 |

^a^ Row percentage ^b^ Column percentage ^c^ 1 household removed from Australian Capital Territory and 0 respondents from Northern Territory
