# Supplementary material for: The role of dwelling type on food expenditure: a cross-sectional analysis of the 2015–2016 Australian Household Expenditure Survey
Source: Public Health Nutr. 2020 Aug 24;24(8):2132–43. doi: 10.1017/S1368980020002785 (PMC8145465; doi:10.1017/S1368980020002785)
Supplement: Supplementary file 1 [file S1368980020002785sup.zip › S1368980020002785sup002.docx]

**Additional file 2:** Number of households with a proportion of total weekly food expenditure equal to one per food category across dwelling types

|  | Separate house | Semi-detached house | Low-rise apartment | High-rise apartment | Total |
| --- | --- | --- | --- | --- | --- |
| Fresh fruits | 0 | 0 | 1 | 0 | 1 |
| Fresh vegetables | 0 | 0 | 0 | 0 | 0 |
| Pre-prepared meals | 2 | 0 | 0 | 0 | 2 |
| Meals in restaurants, hotels and clubs | 2 | 0 | 2 | 1 | 5 |
| Fast food and takeaway | 0 | 2 | 1 | 0 | 3 |
| Total | 4 | 2 | 4 | 1 | 11 |
