# Supplementary material for: The role of dwelling type on food expenditure: a cross-sectional analysis of the 2015–2016 Australian Household Expenditure Survey
Source: Public Health Nutr. 2020 Aug 24;24(8):2132–43. doi: 10.1017/S1368980020002785 (PMC8145465; doi:10.1017/S1368980020002785)
Supplement: Supplementary file 1 [file S1368980020002785sup.zip › S1368980020002785sup003.docx]

**Additional file 3:** Odds ratios of observing a zero proportion and relative proportion ratios

| N=7347 | Fresh fruits | | Fresh vegetables | | Pre-prepared meals | | Meals in restaurants, hotels and clubs | | Fast food and takeaway | |
| --- | --- | --- | --- | --- | --- | --- | --- | --- | --- | --- |
|  | **RPR** | **ci95** | **RPR** | **ci95** | **RPR** | **ci95** | **RPR** | **ci95** | **RPR** | **ci95** |
| Proportion (beta regression) ^a^ |  |  |  |  |  |  |  |  |  |  |
| Semi-detached | 1.037 | 0.980,1.096 | 1.015 | 0.961,1.072 | 0.945 | 0.884,1.011 | 1.171 | 1.085,1.264 | 0.935 | 0.878,0.996 |
| Low-rise apartment | 1.169 | 1.085,1.259 | 1.047 | 0.973,1.127 | 0.928 | 0.844,1.019 | 1.193 | 1.078,1.321 | 1.013 | 0.928,1.107 |
| High-rise apartment | 1.012 | 0.914,1.120 | 0.988 | 0.888,1.100 | 0.859 | 0.750,0.983 | 1.726 | 1.494,1.994 | 0.926 | 0.820,1.046 |
|  | **OR** | **ci95** | **OR** | **ci95** | **OR** | **ci95** | **OR** | **ci95** | **OR** | **ci95** |
| Zero-inflate (logistic regression) ^a^ |  |  |  |  |  |  |  |  |  |  |
| Semi-detached | 0.895 | 0.720,1.112 | 0.970 | 0.771,1.220 | 1.136 | 0.978,1.319 | 0.754 | 0.641,0.886 | 0.776 | 0.646,0.933 |
| Low-rise apartment | 0.829 | 0.647,1.062 | 0.786 | 0.602,1.024 | 1.197 | 0.990,1.449 | 0.750 | 0.612,0.919 | 0.947 | 0.756,1.186 |
| High-rise apartment | 0.587 | 0.393,0.876 | 0.615 | 0.389,0.971 | 1.479 | 1.120,1.953 | 0.434 | 0.316,0.595 | 0.939 | 0.663,1.328 |

RPR = relative proportion ratio; ci95 = 95% confidence interval;  ^a^ Reference group = Separate house; OR = odds ratio - models adjusted for confounders
